# *Coraliomargarita parva* sp. nov., isolated from mangrove sediment and genome-based analysis of the class *Opitutae* revealed five novel families: *Coraliomargaritaceae* fam. nov., *Pelagicoccaceae* fam. nov., *Cerasicoccaeae* fam. nov., *Oceanipulchritudinaceae* fam. nov., and *Alterococcaeae* fam. nov.

**DOI:** 10.3389/fmicb.2023.1202141

**Published:** 2023-06-09

**Authors:** Lingli Min, Wenzhuo Wang, Aharon Oren, Qiliang Lai, Zhaobin Huang

**Affiliations:** ^1^School of Resources and Environmental Sciences, Quanzhou Normal University, Quanzhou, China; ^2^College of Oceanology and Food Science, Quanzhou Normal University, Quanzhou, China; ^3^The Institute of Life Sciences, The Hebrew University of Jerusalem, Edmond J. Safra Campus, Jerusalem, Israel; ^4^Key Laboratory of Marine Genetic Resources, Third Institute of Oceanography, Ministry of Natural Resources, Xiamen, China; ^5^Fujian Province Key Laboratory for the Development of Bioactive Material from Marine Algae, Quanzhou, China

**Keywords:** *Coraliomargarita*, *Opitutae*, genome-based analysis, phylogeny, polyphasic taxonomy, *Puniceicoccaceae*, new families

## Abstract

Members of the class *Opitutae* are widely distributed in various environments such as rice paddy soil, freshwater lakes, seawater, marine sediment, and invertebrate digestive tracts. The class currently consists of two orders, *Opitutales* and *Puniceicoccales*, represented by the families *Opitutaceae* and *Puniceicoccaceae*, respectively, which are primarily delineated on the basis of 16S rRNA gene sequences and limited phenotypic characterizations of a few type strains. The scarcity of 16S rRNA gene and genome sequences generated from the type strains of the class *Opitutae* constrained our understanding of the ecological distribution and adequate resolution of its taxonomy. Here, an *Opitutae* strain designated WMMB3^T^, isolated from a mangrove sediment, was subjected to taxonomic characterization. The 16S rRNA gene of strain WMMB3^T^ shared high sequence similarities with *Coraliomargarita akajimensis* DSM 45221^T^ and *C. sinensis* WN38^T^ of 96.1 and 95.9%, respectively. Phylogenetic analysis suggested that strain WMMB3^T^ formed a monophyletic branch affiliated to the genus *Coraliomargarita*. The average nucleotide identity (ANI) values, digital DNA–DNA hybridization (dDDH) values and average amino acid identity (AAI) values of strain WMMB3^T^ compared between *Coraliomargarita* members were 71.8–72.5, 20.7, and 68.2–68.7%, respectively, indicating that strain WMMB3^T^ represented a novel species of *Coraliomargarita*. The genome of strain WMMB3^T^ was 4.5 Mbp with a DNA G + C content of 56.0%. The respiratory quinone was menaquinone-7. The major fatty acids were iso-C_14:0_, and C_18:1_*ω*9*c*. Based on genomic, phenotypic, and chemotaxonomic characterizations, strain WMMB3^T^ represents a novel species, and *Coraliomargarita parva* sp. nov. is proposed. Additionally, the phylogenomic analysis of more than 500 genomes of the class *Opitutae*, encompassing a majority of uncultivated bacteria and a few type strains, was performed using the Genome Taxonomic Database toolkit (GTDB-Tk) to present adequate resolution of the taxonomy. Combined with 16S rRNA gene sequence phylogeny and genomic relatedness, five novel families retrieved mainly from marine habitats were proposed: *Coraliomargaritaceae* fam. nov., *Pelagicoccaceae* fam. nov., *Cerasicoccaeae* fam. nov., *Oceanipulchritudinaceae* fam. nov., and *Alterococcaeae* fam. nov. AAI values of 58–60% could be considered as the boundary to delineate families of the class *Opitutae*. This study provided a new taxonomic framework of the class *Opitutae* based on the genomic data.

## 1. Introduction

The class *Opitutae* within the phylum *Verrucomicrobiota* is widely distributed in various environments, including rice paddy soil, freshwater lakes, seawater, marine sediment, and digestive tracts of invertebrate hosts, such as marine clamworms, ciliates, and sea cucumbers (Choo et al., 2007). The class currently comprises two orders, *Opitutales* and *Puniceicoccales*, represented by the families *Opitutaceae* and *Puniceicoccaceae*, respectively, primarily classified based on the phylogeny of 16S rRNA gene sequences. Members of the family *Opitutaceae* were mainly retrieved from soil and terrestrial habitats, while those of the family *Puniceicoccaceae* were derived from marine environments (Choo et al., 2007). Nearly 20 species with validly published names were described in the class *Opitutae* (Parte et al., 2020). The scarcity of the 16S rRNA gene sequences and genome sequences generated from the type strains of the class *Opitutae* constrains our understanding of its taxonomy.

*Coraliomargarita*, a genus of the family *Puniceicoccaceae*, order *Puniceicoccales*, is described on the basis of 16S rRNA gene phylogeny and physiological and chemotaxonomic characteristics (Yoon et al., 2007b). Until now, this genus includes two species with validly published names, *Coraliomargarita akajimensis* (Yoon et al., 2007b) and *Coraliomargarita sinensis* (Zhou et al., 2019). Cells of *Coraliomargarita* are Gram-stain-negative, obligately aerobic, coccus-shaped, non-motile, and oxidase- and catalase- positive (Yoon et al., 2007b). The major respiratory quinone is menaquinone-7 (MK-7). The genomic DNA G + C contents calculated from genome sequences are 53.6–54.7%. The predominant cellular fatty acids are C_14:0_, C_18:1_ *ω*9*c*, and C_18:0_ (Yoon et al., 2007b; Zhou et al., 2019). The 16S rRNA gene sequences of *Coraliomargarita* showed low sequence similarities of 88.2–89.2% with *Puniceicoccus vermicola* IMCC1545^T^, which made us question the taxonomic placement of *Coraliomargarita* into the family *Puniceicoccaceae*.

In this study, a novel strain designated WMMB3^T^, isolated from a mangrove sediment, was found to represent a novel species of the genus *Coraliomargarita*. This study aimed to determine the taxonomic status of strain WMMB3^T^ using a polyphasic taxonomic approach. Additionally, the phylogeny of the class *Opitutae* members was investigated based on the currently available genomes including a majority of uncultivated bacteria obtained using metagenomic assembled genomes (MAGs) and single cell genomes (SAGs) and a few type strains. This study provided new insights into the taxonomy of the class *Opitutae.*

## 2. Materials and methods

### 2.1. Strain isolation and cultivation

Strain WMMB3^T^ was isolated from a mangrove sediment, collected from a mangrove preservation area (E 118.699^o^, N 24.937^o^) in Quanzhou on Sep. 15, 2022. About 1 g of sediment was added to 9 mL sterile natural seawater and vigorously shaken to make a suspension. Serial 10-fold dilutions were spread on 2216E culture plates (5 g/L peptone, 1 g/L yeast extract, 15 g/L agar, 1 L natural seawater). The plates were maintained at 30°C for 14  days. Strain WMMB3^T^ was picked and streaked twice on Marine Broth 2216 (MB, BD) agar plates to obtain a pure culture. The strain was routinely cultured in MB and on MB agar plates at 30°C, and stored at -80°C in 20% glycerol (v/v). Strain WMMB3^T^ was deposited in the Marine Culture Collection of China (MCCC 1K08426^T^) and the Korean Collection for Type Cultures (KCTC 92914^T^).

For comparative purpose, the two type strains of species of the genus *Coraliomargarita*, *C. akajimensis* DSM 45221^T^ (=04OKA010-24^T^ = MCCC 1A12044^T^) and *C. sinensis* WN38^T^ (=KCTC 62602^T^ = MCCC 1H00313^T^) were used as reference strains.

### 2.2. Phylogenetic analysis of 16S rRNA gene sequences

The genomic DNA of strain WMMB3^T^ was extracted using a bacterial genomic DNA extraction kit (Saibaisheng, Shanghai, China). The 16S rRNA gene of strain WMMB3^T^ was PCR-amplified in a 50 μL amplification system (*Ex* Taq, TaKaRa) using primers Eub27F and 1492R (DeLong, 1992). The PCR product of ~1.5 kb was detected using 1.5% agarose gel electrophoresis, and the sequence was determined by using an ABI 3730 sequencer (Sanger sequencing).

The closest type strains of strain WMMB3^T^ were identified by using the EzBioCloud (Yoon et al., 2017a), *nr* database in GenBank^1^ and SILVA 132 database (Yilmaz et al., 2014). The 16S rRNA gene sequences were aligned using ClustalW, and neighbor-joining and maximum-likelihood phylogenetic trees were reconstructed using MEGA 7.0 (Kumar et al., 2016). The models used in neighbor-joining (NJ) and maximum-likelihood (ML) were maximum composite likelihood (MCL) and K2 + G + I, respectively. The topology was evaluated based on 1,000 bootstrap replicates for the NJ and ML methods.

### 2.3. Genome sequencing and annotation

The draft genome sequence of strain WMMB3^T^ was determined using the Illumina Hiseq platform following the manufacturer’s instruction (Shanghai Majorbio Bio-Pharm Technology Co., Ltd., Shanghai, China). Paired-end reads (PE reads) of 2 × 151 bp were quality checked (q > 20 and length > 50 bp) using sickle,^2^ and assembled into contigs using SPAdes v. 3.8.0 (Bankevich et al., 2012) with a serial of *k* values (21, 33, 55, 77, 99, 127) and -*careful* flag. The assembled contigs with <1 kbp were removed from the following analysis. The prediction of functional genes was performed using prodigal (Hyatt et al., 2010), and also annotated using the RAST server (Aziz et al., 2008).

### 2.4. Phylogenomic analysis

The genomes of type strains affiliated to the class *Opitutae* were used to reconstruct a phylogenomic tree using GTDB-Tk v1.3.0 (Parks et al., 2018) with 120 conserved concatenated proteins referred to as the Bac120 set. The tree was visualized using the Interactive Tree of Life (iTOL) online (Letunic and Bork, 2007).

### 2.5. Genomic relatedness

The average nucleotide identity (ANI) values were calculated using ANI Calculator (Yoon et al., 2017b). The digital DNA–DNA hybridization (dDDH) values were calculated using the online Genome-to-Genome Distance Calculator (GGDC, version 3.0) (Meier-Kolthoff et al., 2013). The average amino acid identity (AAI) values were calculated using CompareM v0.1.2.^3^

### 2.6. Characterization of cell morphology and physiology

Gram staining of strain WMMB3^T^ was conducted by using a Gram staining kit (Solarbio Co, Beijing). Catalase activity was tested by using 3% (v/v) H_2_O_2_ solution. Oxidase activity was tested by using an oxidase reagent (N,N,N,N-tetramethyl-p-phenylenediamine dihydrochloride, bioMérieux, France). To observe the growth temperature range, strains were streaked on MB agar plates and maintained at various temperatures (10, 15, 20, 25, 30, 35, 40, and 45°C) for 7 days. The ability of degrading substrates, including soluble starch (1%, w/v), skimmed milk (1%, w/v), cellulose (1%, w/v), Tween 20 (1%, v/v), Tween 40 (1%, v/v), Tween 60 (1%, v/v), and Tween 80 (1%, v/v) were tested by streaking the strains onto the MB agar plates with each substrate (Huang et al., 2021). Additional physiological and biochemical characteristics were tested by using three API strips, including API ZYM, API 20NE and API 20E (bioMérieux product, France) according to the manufacturer’s instructions.

### 2.7. Chemotaxonomic characteristics

For cellular fatty acids composition analysis, strain WMMB3^T^ and reference strains were cultured in MB at 30°C for 5 days and cells were harvested by centrifugation at 8,000 rpm. The cellular fatty acids were extracted and identified by gas chromatography following the standard MIDI protocol (Sherlock Microbial Identification System, version 6B). The respiratory quinone of strain WMMB3^T^ was extracted using chloroform/methanol (2:1, v/v), and identified and quantified using reversed phase high-performance liquid chromatography as described previously (Komagata and Suzuki, 1987).

### 2.8. Phylogenomic analysis of the class *Opitutae*

The genomes affiliated to the class *Opitutae* (until Nov. 17. 2022) were downloaded from the genome portal in GenBank.^4^ The quality of these genomes was evaluated using CheckM v1.0.12 (Parks et al., 2015), and the genomes with > = 90% completeness and < =5% contamination were maintained for the subsequent phylogenomic study (Supplementary Table S1). A phylogenomic tree of the high-quality genomes was inferred using a concatenated alignment of Bac120 with GTDB-Tk v. 1.3.0 by using FastTree (Parks et al., 2018). The tree was visualized using the iTOL online (Letunic and Bork, 2007).

### 2.9. Phylogeny of 16S rRNA gene sequences of the class *Opitutae*

The 16S rRNA gene sequences were identified and extracted from all downloaded *Opitutae* genomes using RNAmmer with default parameters (Lagesen et al., 2007). The 16S sequences exhibiting >1,200 bp in length, < 8 polymers and no ambiguous bases were selected by using screen.seqs command in mothur (Schloss et al., 2009). The representative sequences were identified by using RDP Classifier (Wang et al., 2007) and aligned using ClustalW. Phylogenetic trees of 16S rRNA gene sequences were constructed using NJ and ML in MEGA 7.0 (Kumar et al., 2016). The trees were finally visualized using the Interactive Tree of Life (iTOL) online (Letunic and Bork, 2007).

## 3. Results and discussion

### 3.1. Phylogeny of the 16S rRNA gene

**Figure 1:**
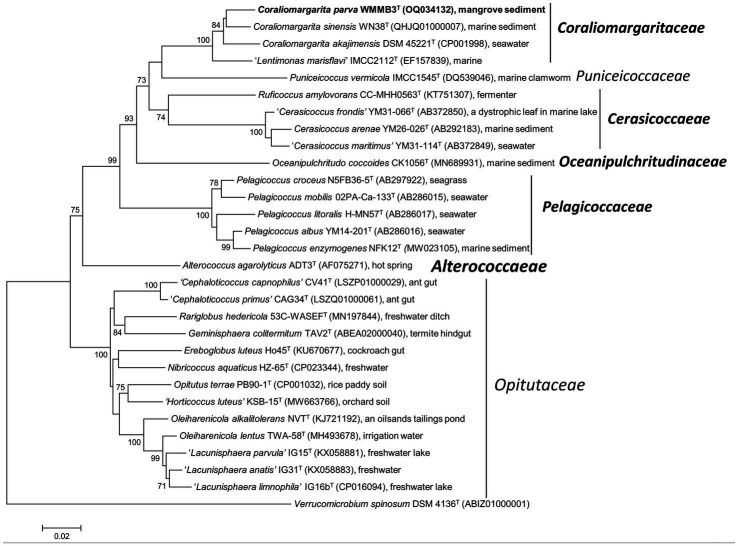
Neighbor joining phylogenetic tree of strain WMMB3^T^ and its close relatives constructed based on 16S rRNA gene sequences. *Coraliomargarita parva* WMMB3^T^ is marked bold. Bootstrapping was carried out with 1,000 replicates. Node values below 70% are not shown. *Verrucomicrobium spinosum* DSM 4136^T^ was selected as the outgroup. Bar, 0.02 means the nucleotide substitution per position. The newly described families are marked bold.

Phylogeny of the 16S rRNA gene sequences showed that strain WMMB3^T^ belonged to the genus *Coraliomargarita* of the class *Opitutae*, and formed a monophyletic branch with *C. akajimensis* DSM 45221^T^ and *C. sinensis* WN38^T^ (Figure 1; Supplementary Figure S1), sharing sequence similarities of 96.1 and 95.9%, respectively. The 16S rRNA gene of strain WMMB3^T^ had <88.3% sequence similarity with *Puniceicoccus vermicola* IMCC1545^T^ and the other species with validly published names within the class *Opitutae*. The low sequence similarity of its 16S rRNA gene with its closest relatives demonstrated that strain WMMB3^T^ may represent a novel species.

Phylogeny of 16S rRNA gene of the type strains also revealed that nine genus-level members formed a highly supported clade (bootstrap values of 99–100%), belonging to the family *Opitutaceae* (Figure 1; Supplementary Figure S1). They were *Opitutus* (type genus), ‘*Cephaloticoccus*’, *Rariglobus*, *Geminisphaera*, *Ereboglobus*, *Nibricoccus*, “*Horticoccus*”, *Oleiharenicola*, and “*Lacunisphaera*”. These members are mainly terrestrial in origin, for instance, soil, freshwater lakes and the guts of terrestrial invertebrates (Figure 1). *Alterococcus agarolyticus* ADT3^T^, currently classified in the family *Opitutaceae* (Yoon et al., 2017a; Parte et al., 2020), formed a distant branch with *Optitutus* and other members of the family *Opitutaceae*, and could therefore be assigned to a novel family. Thus, a new family *Alterococcaeae* fam. nov. is proposed to accommodate this genus *Alterococcus*. *A. agarolyticus* ADT3^T^ is a halophilic thermophilic bacterium, isolated from an intertidal hot spring (Shieh and Jean, 1998), a habitat different from that of the other members of the family *Opitutaceae*. The 16S rRNA gene of *A. agarolyticus* ADT3^T^ shared 88.3 and 84.5% sequence similarity with *Opitutus terrae* PB90-1^T^ and *P. vermicola* IMCC1545^T^, respectively.

Seven other genera, including *Pelagicoccus*, *Oceanipulchritudo*, *Cerasicoccus*, *Ruficoccus, Puniceicoccus* (type genus), *Coraliomargarita*, and “*Lentimonas*” grouped into a coherent clade. Members of these seven genera are primarily marine in origin (Figure 1). However, these groups are need to be re-classified into different families based on phylogenomic analysis of the genome sequence and genomic relatedness described below.

### 3.2. Genome-based analysis

The draft genome size of strain WMMB3^T^ was 4,497,538 bp on 27 assembled contigs (>1 kb in length), which was larger than that of *C. akajimensis* DSM 45221^T^ with 3,750,771 bp and that of *C. sinensis* WN38^T^ with 3,569,655 bp. The genomic DNA G + C content of strain WMMB3^T^ was 56.0% (Table 1), which was also higher than *C. akajimensis* DSM 45221^T^ (53.6%) and *C. sinensis* WN38^T^ (54.7%).

**Table 1:** Differential characteristics of strain WMMB3^T^ compared to close relatives of the genus *Coraliomargarita*.

Characteristics	1	2	3
Growth temperature range (Optimal ^°^C)	15–40 (30)	20–35 (30)	20–40 (35)
*α*-Chymotrypsin	+	w	
Nitrate reduction	+	+	−
Hydrolysis of aesculin	−	+	+
Esterase (C4)	+	w	w
Esterase lipase (C8)	−	−	w
Acid phosphatase	+	+	−
Naphthol-AS-BI-phosphohydrolase	+	w	+
*α*-Galactosidase	w	−	−
*β*-Galactosidase	+	w	−
*α*-Glucosidase	w	−	−
*β*-Glucosidase	w	−	−
*α*-Mannosidase	−	−	+
Acid can be produced from			
Glucose	+	+	−
Mannitol	w	−	−
Amygdalin, arabinose	+	−	−
Genome size (bp)	4,497,538	3,750,771	3,569,655
DNA G + C content (%)	56.0	53.6	54.7

1. Strain WMMB3^T^, 2. *C. akajimensis* DSM 45221^T^, 3. *C. sinensis* WN38^T^. +, positive; −, negative; w, weak positive.

The average nucleotide identity (ANI) values of WMMB3^T^ compared against *C. akajimensis* DSM 45221^T^ and *C. sinensis* WN38^T^ were 71.8 and 72.5%, respectively. The digital DNA–DNA hybridization (dDDH) values of WMMB3^T^ against *C. akajimensis* DSM 45221^T^ and *C. sinensis* WN38^T^ were both 20.7%. These values were below the threshold values of prokaryotic species definition (95–96% ANI and 70% DDH) (Richter and Rosselló-Móra, 2009), which strongly supported that strain WMMB3^T^ belonged to a novel species. The average amino acid identity (AAI) values of strain WMMB3^T^ against *C. akajimensis* DSM 45221^T^ and *C. sinensis* WN38^T^ were 68.2 and 68.7%, respectively, which exceeded the cutoff of the same genus (65–95% AAI) (Konstantinidis et al., 2017), suggesting that WMMB3^T^ belonged to the genus *Coraliomargarita*.

**Figure 2:**
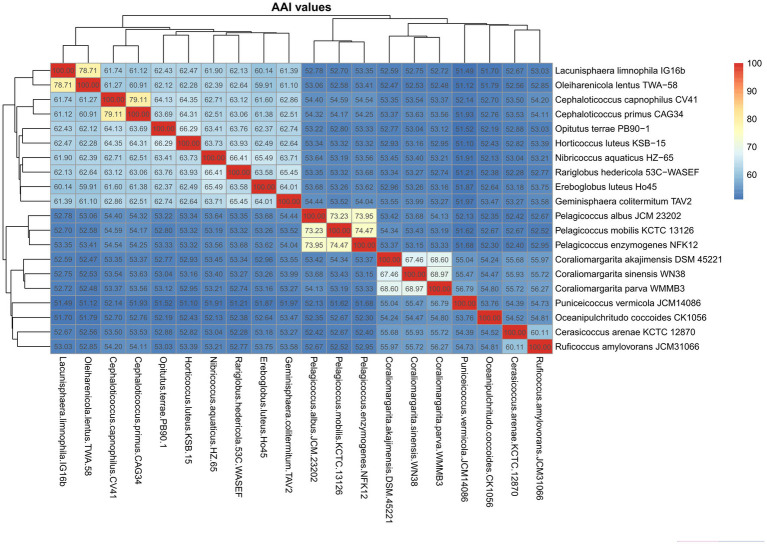
Heatmap showing the AAI values calculated among the species of the class *Opitutae*.

The AAI values calculated among the 10 species of the family *Opitutaceae* were 59.9–79.1% (Figure 2). The AAI values of the *Pelagicoccus* species and the *Coraliomargarita* species were 73.2–74.5% and 67.5–69.0%, respectively, and were clearly separated from other members of the class *Opitutae*. The two genera could be considered two families, as also supported by phylogenetic analysis. *Cerasicoccus arenae* KCTC 12870^T^ had 60.1% AAI with *Ruficoccus amylovorans* JCM 31066^T^, which could be united into the same family. The above four genera had <60% AAI with *P. vermicola* JCM14086^T^ and *Oceanipulchritudo coccoides* CK1056^T^. Thus, AAI value of 60% could be regarded the threshold to differentiate these genera. The boundary of AAI values to divide these species into two orders *Opitutales* and *Puniceicoccales* is not distinct. To be congruent with GTDB taxonomy (Chaumeil et al., 2019), we suggested to unite the two orders.

### 3.3. Phylogenomic analysis

**Figure 3:**
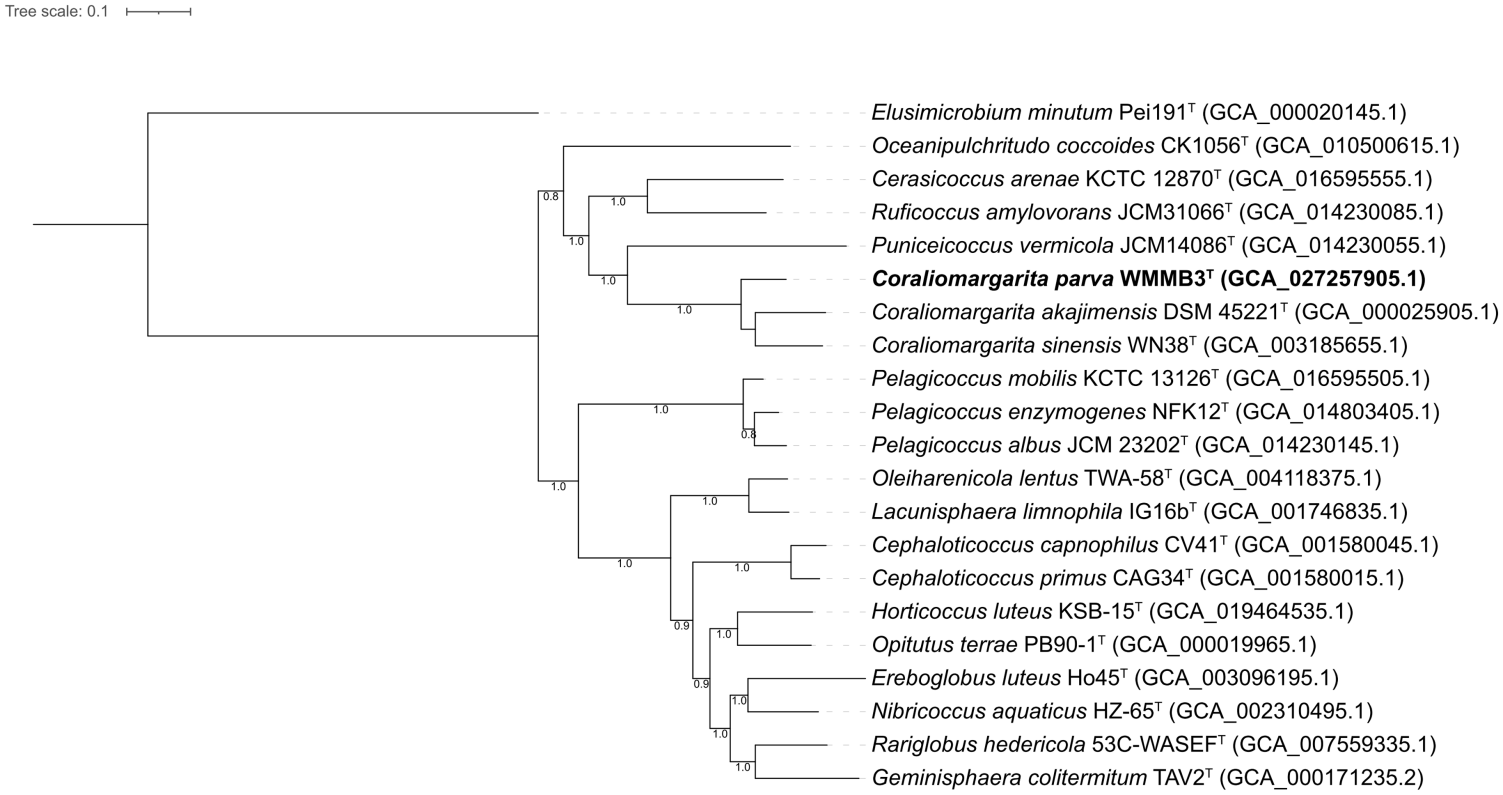
Phylogenomic tree constructed based on 120 bacterial conserved concatenated proteins showing the relationship between strain WMMB3^T^ and the type strains of the class *Opitutae*. The tree was rooted by *Elusimicrobium minutum* Pei191^T^. *Coraliomargarita parva* WMMB3^T^ is marked bold. The bootstrap values of nodes greater than 0.7 are displayed. Bar, 0.1 means the amino acid substitutions per position.

A phylogenomic tree including the type strains affiliated to the class *Opitutae* was constructed using GTDB-Tk v.1.3.0 (Figure 3), a powerful tool to resolve the taxonomic position of the bacterial and archaeal genomes based on the Bac120 set (Chaumeil et al., 2019). The phylogenomic tree showed that strain WMMB3^T^ formed a highly supported and monophyletic clade with *C. akajimensis* DSM 45221^T^ and *C. sinensis* WN38^T^, supporting that strain WMMB3^T^ represented a novel species within the genus *Coraliomargarita* (Figure 3). Moreover, we found that *Pelagicoccus* members formed a distinct group, which should be regarded as a novel family, but not as a member in the family *Puniceicoccaceae* (Yoon et al., 2017a; Parte et al., 2020). Thus, *Pelagicoccaceae* fam. nov. was proposed to accommodate the genus *Pelagicoccus*. Members of *Pelagicoccus* were found in seawater, marine sediment and seagrass-associated environments (Figure 1).

### 3.4. Morphological and physiological characteristics

Colonies of strain WMMB3^T^ on agar plates cultured at 30°C for 5 days were small and round. Cells were Gram-stain-negative, aerobic, and coccus-shaped. Oxidase and catalase activities were positive, as for *C. akajimensis* DSM 45221^T^ and *C. sinensis* WN38^T^. The growth temperature range of strain WMMB3^T^ was 15–40°C with optimal temperature of 30°C (Table 1). Growth was not observed at 10 and 45°C.

Hydrolysis of Tween 20, Tween 40, Tween 60 and Tween 80 was not observed for strain WMMB3^T^, as for *C. akajimensis* DSM 45221^T^ and *C. sinensis* WN38^T^. Strain WMMB3^T^ cannot degrade soluble starch, CMC, and skimmed milk, similar to *C. akajimensis* DSM 45221^T^ and *C. sinensis* WN38^T^. Nitrate can be reduced to nitrite for strain WMMB3^T^ and *C. akajimensis* DSM 45221^T^, but different from *C. sinensis* WN38^T^ (Table 1 and species description). Production of indole is positive and hydrolysis of gelatin is negative. The other phenotypic characteristics are summarized in the species description.

### 3.5. Chemotaxonomic characteristics

The sole respiratory quinone of strain WMMB3^T^ was menaquinone 7 (MK-7), similar to *C. akajimensis* DSM 45221^T^ (Yoon et al., 2007b) and *C. sinensis* WN38^T^ (Zhou et al., 2019). The major fatty acids (>10%) of strain WMMB3^T^ were iso-C_14:0_ (23.1%), and C_18:1_ *ω*9*c* (19.0%), similar to *C. sinensis* WN38^T^ and *C. akajimensis* DSM 45221^T^. However, there were differences in the relative abundance of some fatty acids (Table 2).

**Table 2:** Cellular fatty acid composition of strain WMMB3^T^ compared to its close relatives of the genus *Coraliomargarita.*

Fatty acid	1	2	3
Saturated			
C_12:0_	1.4	–	–
C_14:0_	4.5	**16.9**	**5.4**
C_16:0_	4.4	**5.1**	3.5
C_17:0_	3.0	tr	3.2
C_18:0_	**7.5**	**13.1**	**10.2**
C_19:0_	1.8	1.2	5.0
C_20:0_	4.4	**6.8**	2.2
iso-C_10:0_	1.6	4.2	1.3
iso-C_11:0_	–	2.0	–
iso-C_12:0_	2.6	–	–
iso-C_14:0_	**23.1**	**9.9**	**16.7**
iso-C_16:0_	2.8	2.2	3.4
iso-C_18:0_	3.6	tr	4.3
anteiso-C_13:0_	1.4	1.2	tr
anteiso-C_15:0_	4.5	2.4	**11.1**
anteiso-C_17:0_	1.0	tr	2.2
*Hydroxy*			
C_10:0_ 3-OH	1.4	–	–
C_12:0_ 3-OH	1.7	2.7	1.1
C_16:0_ 3-OH	–	1.1	–
iso-C_11:0_ 3-OH	tr	1.3	–
iso-C_14:0_ 3-OH	–	–	2.2
Unsaturated			
C_17:1_ *ω*8*c*	1.4	tr	4.4
C_18:1_ *ω*9*c*	**19.0**	**22.6**	**15.5**
*Summed features*^†^			
8	tr	1.1	1.2

1. strain WMMB3^T^; 2. *C. akajimensis* DSM 45221^T^. 3. *C. sinensis* WN38^T^. All data were obtained from this study. –, not detected; tr, trace <1%.

† Summed Features are fatty acids that cannot be resolved reliably from another fatty acid using the chromatographic conditions chosen. The MIDI system groups these fatty acids together as one feature with a single percentage of the total. Summed feature 8 comprised C_18:1_ *ω*7*c* and/or C_18:1_ *ω*6*c.*The major fatty acids (>10%) are marked bold. The values below 10% are not marked bold.

### 3.6. Phylogenomic analysis of *Opitutae* genomes

**Figure 4:**
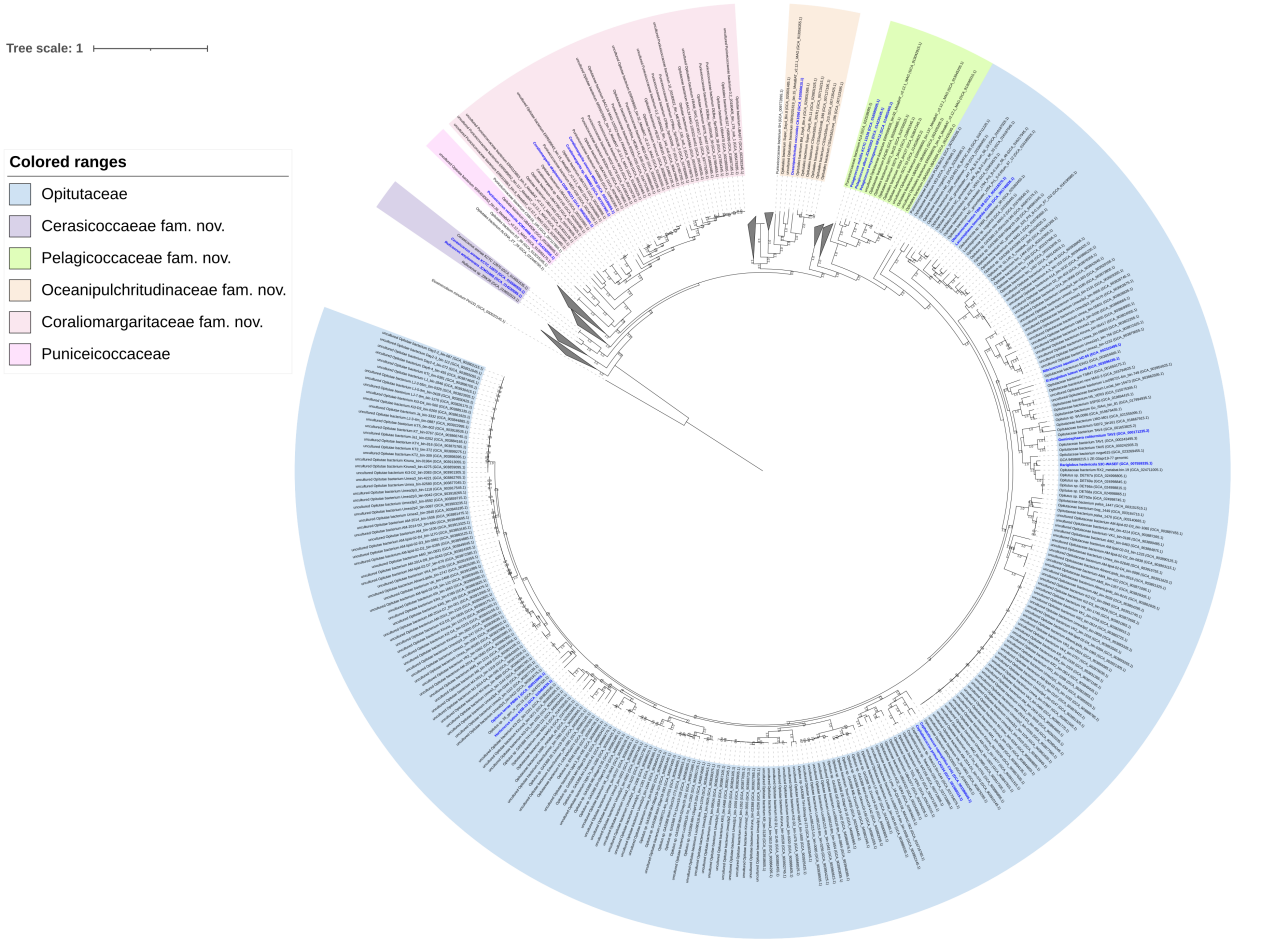
Phylogenomic tree constructed based on 120 bacterial conserved concatenated proteins showing the relationship of the 546 genomes affiliated to the class *Opitutae*. The tree was rooted by *Elusimicrobium minutum* Pei191^T^. The bootstrap values of the node greater than 0.7 are displayed. Bar, 1 means the amino acid substitutions per position. Type strains are marked blue. The clades without cultivated representatives were collapsed.

A total of 1,105 genomes affiliated to the class *Opitutae* were downloaded from the Genome portal of GenBank (Supplementary Table S1). Genomes with ≥90% completeness and ≤ 5% contamination were used, which were verified to perform accurate phylogenetic analysis. The phylogenomic tree including a total of 546 genomes that met the above standards was inferred by using GTDB-Tk (Bowers et al., 2017; Figure 4; Supplementary Figure S2).

Strain WMMB3 was placed in the lineage including *C. sinensis* WN38^T^ and *C. akajimensis* DSM 45221^T^. This lineage containing *Coraliomargarita* should be considered as a novel family of the class *Opitutae*. Thus, *Coraliomargaritaceae* fam. nov. was proposed. The AAI values of the *Coraliomargaritaceae* members were 59.7–100%. The genome size of *Coraliomargaritaceae* members was 1.2–4.7 Mbp with a genomic G + C content of 38.5–56.4%.

*Cerasicoccus arenae* KCTC 12870^T^, *Ruficoccus amylovorans* JCM 31066^T^ and *Ruficoccus* sp. ZRK36 formed a deeply branched clade, which should be recognized as a novel family (Figure 3). The AAI values among the three strains were 59.3–78.9%, demonstrating they belonged to two different genera of the same family (Konstantinidis et al., 2017). Thus, a novel family named *Cerasicoccaceae* fam. nov. was proposed to accommodate the genera *Cerasicoccus* and *Ruficoccus*. The genome size was 3.8–4.5 Mbp with genomic G + C content of 52.6–60.3%. Members were found in marine sediments, seawater, deep-sea cold seeps and liquid fertilizer of a fermenter in a greenhouse facility (Figure 1).

*Puniceicoccus vermicola* JCM14086^T^ and two uncultivated bacterial genomes formed a tight clade, constituting the members of the family *Puniceicoccaceae*. The genome size of this family was 2.6–5.2 Mbp with genomic G + C content of 54.3–68.6%. The AAI values computed with the three genomes were 61.2–75.1%.

*Oceanipulchritudo coccoides* CK1506^T^ grouping with eight uncultivated bacterial genomes formed a monophyletic clade, which was distant from the family *Puniceicoccaceae*. *Oceanipulchritudo coccoides* should be classified in a novel family, and thus, *Oceanipulchritudinaceae* fam. nov. was proposed. The AAI values computed with the uncultivated genomes were 58.4–99.3%. The genome size of members of the *Oceanipulchritudinaceae* was 3.5–5.3 Mbp with genomic G + C content of 54.0–63.8%.

*Pelagicoccus*, currently including five species with validly published names (Parte et al., 2020), also represented a novel family, which was confirmed by phylogenomic analysis based on a few type strains (Figure 3). The genome size was 3.1–7.5 Mbp with genomic G + C content of 46.1–56.8%. The AAI values of *Pelagicoccaceae* genomes including uncultivated bacteria were 60.5–99.9%.

The differential characteristics of the novel families compared to *Opitutaceae* and *Puniceicoccaceae* were summarized in Table 3. A large number of clades (collapsed lineages) lacking species with validly published names or cultivated strains may represent novel families of the class *Opitutae*. The bacteria in these clades are waiting cultivation, taxonomic characterization and naming.

**Table 3:** Differential characteristics compared among the members in the class *Opitutae.*

Characteristics	*Opitutaceae*	*Puniceicoccaceae*	*Coraliomargaritaceae*	*Cerasicoccaceae*	*Pelagicoccaceae*	*Oceanipulchritudinaceae*	*Alterococcaeae* †
Type genus	*Opitutus*	*Puniceicoccus*	*Coraliomargarita*	*Cerasicoccus*	*Pelagicoccus*	*Oceanipulchritudo*	*Alterococcus*
Type species	*Opitutus terrae*	*Puniceicoccus vermicola*	*Coraliomargarita akajimensis*	*Cerasicoccus arenae*	*Pelagicoccus mobilis*	*Oceanipulchritudo coccoides*	*Alterococcus agarolyticus*
Motile by flagellum	+^a^	−^b^	−^c^	−^d^	+^e^	+^f^	+
Aerobic growth	Obligately anaerobic^a^	Facultatively anaerobic^b^	Obligately aerobic^c^	Obligately aerobic^d^	Obligately aerobic or facultatively anaerobic^e^	Obligately aerobic^f^	Facultatively anaerobic
Catalase activity	−^a^	−^b^	+	+^d^	v^e^	−^f^	+
Oxidase activity	−^a^	−^b^	+	+^d^	+^e^	+^f^	+
Reduction of nitrate to nitrite	+^a^	−^b^	v	−^d^	−^e^	−^f^	ND
Genome Size (Mbp)	2.1–8.4	3.6–5.2	1.2–4.7	3.8–4.5	3.1–7.5	3.5–5.3	ND
DNA G + C content (%)	42.1–70.0	53.6–56.0	38.5–56.4	52.6–60.3	46.1–56.8	54.0–63.8	65.5–67
AAI values	ND	60.9–75.1	59.7–100	59.3–78.9	60.5–99.9	58.4–99.3	ND
Isolation habitats	Terrestrial ecosystems	Seawater, marine sediment	Marine sediment	Seawater, marine sediment, liquid fertilizer	Seawater, seagrass, marine sediment	Marine sediment	Hot spring

Data were taken from

a Chin et al. (2001).

b Choo et al. (2007).

c Yoon et al. (2007b).

d Yoon et al. (2007a).

e Yoon et al. (2007c).

f Feng et al. (2020).

† Data were taken from (Shieh and Jean, 1998). ND, not determined. v, variable. Cells of all members are cocci. The genome sizes and DNA G + C contents were calculated based on type strains and the uncultivated genomes.

### 3.7. Phylogenetic analysis based on the 16S rRNA gene of *Opitutae*

**Figure 5:**
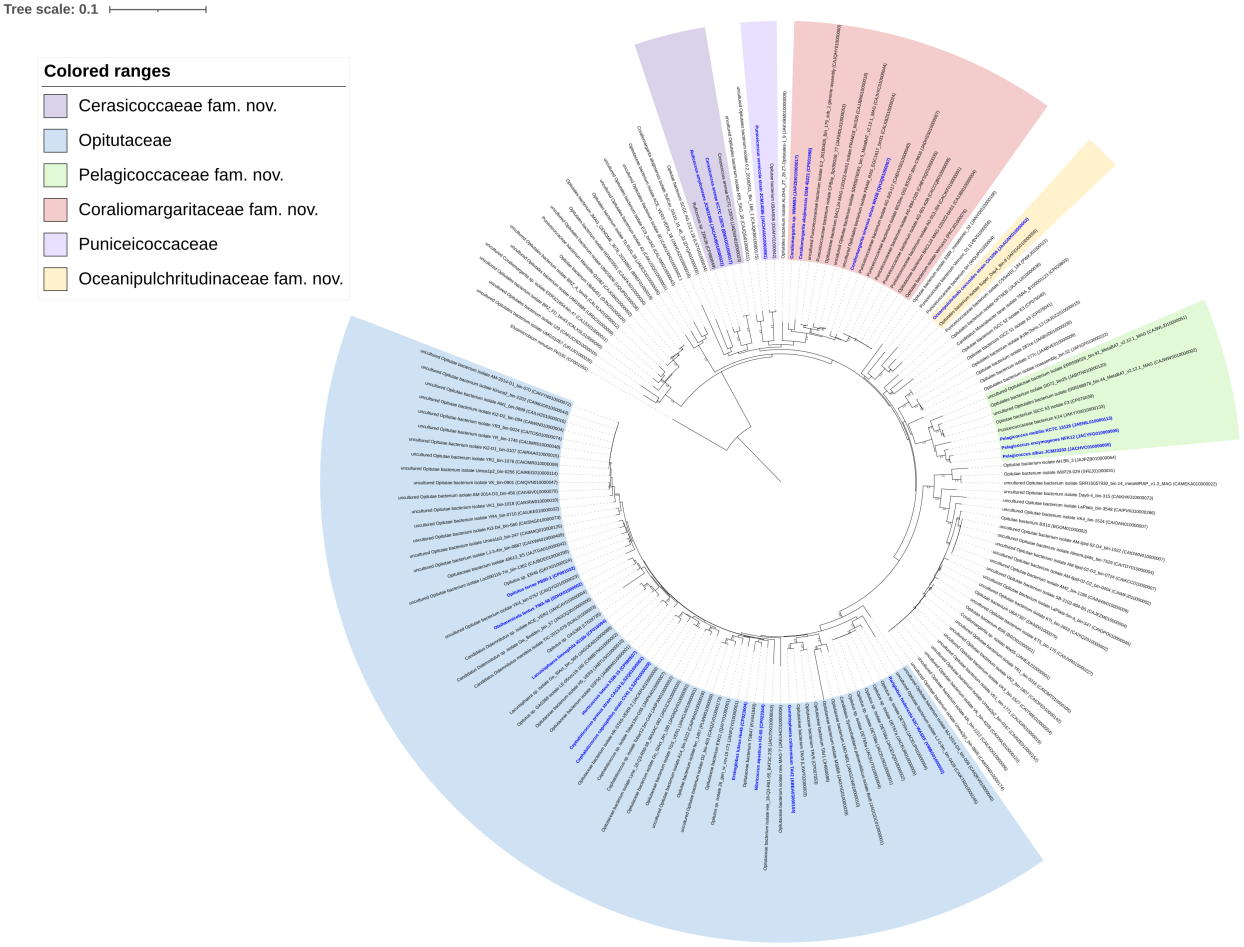
Phylogenetic analysis of high-quality 16S rRNA gene sequences of the class *Opitutae*. The tree was rooted by *Elusimicrobium minutum* Pei191^T^. Type strains are marked blue. The bootstrap values of nodes greater than 0.7 are displayed. Bar, 0.1 means the nucleotide substitution per position.

A total of 156 high-quality 16S rRNA gene sequences with > = 1,200 bp in length were retrieved from 1,105 *Opitutae* genomes. The 16S rRNA sequences were not frequently extracted from MAGs or SAGs, resulting in a smaller dataset than the genomic data used in phylogenomic tree. The phylogeny of 16S rRNA gene sequences revealed similar topology (Figure 5) with the phylogenomic analysis based on a few type strains (Figure 3) and uncultivated bacteria (Figure 4). *Pelagicoccus* is a phylogenetically distant group from the members of the family *Puniceicoccaceae* and represented a novel family (Figure 5). Similar to the genus *Pelagicoccus, O. coccoides* is phylogenetically distant from other members of the class *Opitutae*, and should be classified in a novel family. *C. arenae* KCTC 12870^T^ and *R. amylovorans* JCM 31066^T^ formed a deeply branched clade. They are phylogenetically related with the members of the family *Puniceicoccaceae*, and phylogenomic analysis could warrant the creation of a novel family (Figures 3, 4). The majority of clades presented in Figure 4 lacked cultivated representatives.

## 4. Conclusion

Based on the genomic, phenotypic, and chemotaxonomic characteristics, strain WMMB3^T^ represented a novel species of the genus *Coraliomargarita*, for which the name *Coraliomargarita parva* sp. nov. was proposed. The type strain is WMMB3^T^ (= MCCC 1K04284^T^ = KCTC 92914^T^). Based on the phylogenetic analysis of whole genome sequences and 16S rRNA genes affiliated to the class *Opitutae*, five novel families *Coraliomargaritaceae* fam. nov., *Pelagicoccaceae* fam. nov., *Cerasicoccaeae* fam. nov., *Oceanipulchritudinaceae f*am. nov., and *Alterococcaeae* fam. nov., were proposed. AAI values of 58–60% could be considered the threshold for delineating families of the class *Opitutae*. This study provided a new taxonomic framework of the class *Opitutae* based on genomic data.

### 4.1. Description of *Coraliomargarita parva* sp. nov.

#### 4.1.1. *Coraliomargarita parva* (par’va. L. fem. adj. *parva*, small)

Colonies on MB agar plates cultured for 5 days at 30°C are less than 1 mm, small and round. Cells are Gram-stain-negative and coccus-shaped. Catalase-positive and oxidase-positive. Growth occurs between 15 and 40°C with an optimum at 30°C. Nitrate can be reduced to nitrite. Production of indole is positive, and hydrolysis of aesculin and gelatin is negative. Positive for alkaline phosphatase, acid phosphatase, esterase (C4), naphthol-AS-BI-phosphohydrolase, and *β*-galactosidase; weakly positive for *α*-galactosidase, *α*-glucosidase, and *β*-glucosidase. Acid can be produced from glucose, amygdalin, mannitol and arabinose. The genome size of the type strain is 4.5 Mbp with a DNA G + C content of 56.0%. The respiratory quinone is menaquinone-7 (MK-7). The major fatty acids are iso-C_14:0_ and C_18:1_ *ω*9*c*.

The type strain is WMMB3^T^ (= MCCC 1K08426^T^ = KCTC 92914^T^), isolated from a mangrove sediment collected from a mangrove preservation area in Quanzhou Bay.

The GenBank/EMBL/DDBJ accession number of the 16S rRNA gene sequence and draft genome sequence of strain WMMB3^T^ are Q034132 and JAPZEI000000000, respectively.

### 4.2. Taxonomic consequences: new families

#### 4.2.1. Description of *Coraliomargaritaceae* fam. nov.

*Coraliomargaritaceae* (Co.ra.li.o.mar.ga.ri.ta.ce’ae. N.L. fem. n. *Coraliomargarita*, a bacterial genus; *−aceae*, ending to denote a family; N.L. fem. pl. n. *Coraliomargaritaceae*, the *Coraliomargarita* family).

The description is the same as for the genus *Coraliomargarita* (Yoon et al., 2007b), the type genus and the sole genus in this family. Members are all found in marine sediment and seawater. Delineation of the family is mainly determined by phylogeny of 16S rRNA gene, genomic relatedness and phylogenomic analysis. The genome size is 1.2–4.7 Mbp with genomic G + C content of 38.5–56.4%.

#### 4.2.2. Description of *Pelagicoccaceae* fam. nov.

*Pelagicoccaceae* (Pe.la.gi.coc.*ca.*ce’ae. N.L. masc. n. *Pelagicoccus*, a bacterial genus; *−aceae*, ending to denote a family; N.L. fem. pl. n. *Pelagicoccaceae*, the *Pelagicoccus* family).

The description is the same as for the genus *Pelagicoccus* (Yoon et al., 2007c), the type and the sole genus in this family. Delineation of the family is mainly determined by phylogeny of 16S rRNA gene, genomic relatedness and phylogenomic analysis. The genome size is 3.1–7.5 Mbp with genomic G + C content of 46.1–56.8%.

#### 4.2.3. Description of *Oceanipulchritudinaceae* fam. nov.

*Oceanipulchritudinaceae* (O.ce.a.ni.pul.chri.tu.di.na.ce’ae. N.L. fem. n. *Oceanipulchritudo*, a bacterial genus; *−aceae*, ending to denote a family; N.L. fem. pl. n. *Oceanipulchritudinaceae*, the *Oceanipulchritudo* family).

The description is the same as for the genus *Oceanipulchritudo* (Feng et al., 2020), the type and the sole genus in this family. Delineation of the family is mainly determined by phylogeny of 16S rRNA gene, genomic relatedness and phylogenomic analysis. The genome size is 3.5–5.3 Mbp with genomic G + C content of 54.0–63.8%.

#### 4.2.4. Description of *Cerasicoccaceae* fam. nov.

*Cerasicoccaceae* (Ce.ra.si.coc.*ca.*ce’ae. N.L. masc. n. *Cerasicoccus*, a bacterial genus; *−aceae*, ending to denote a family; N.L. fem. pl. n. *Cerasicoccaceae*, the *Cerasicoccus* family).

Cells are Gram-stain negative, cocci, and obligately aerobic. Cells lack flagella and are non-motile. Spores are not formed. Catalase- and oxidase-positive. The major respiratory quinone is MK-7. Predominant cellular fatty acids are C_14:0_ and C_18:1_ *ω*9*c*. It includes two genera *Cerasicoccus* and *Ruficoccus*. Phylogeny of 16S rRNA gene, genomic relatedness and phylogenomic analysis indicate that *Cerasicoccusaceae* should be separated from the family *Puniceicoccaceae.* The genome size was 3.8–4.5 Mbp with genomic G + C content of 52.6–60.3%. The type genus is *Cerasicoccus*.

#### 4.2.5. Description of *Alterococcaeae* fam. nov.

*Alterococcaeae* (Al.te.ro.coc.*ca.*ce’ae. N.L. masc. n. *Alterococcus*, a bacterial genus; *−aceae*, ending to denote a family; N.L. fem. pl. n. *Alterococcaeae*, the *Alterococcus* family).

The description is the same as for the genus *Alterococcus* (Shieh and Jean, 1998), the type and sole genus in this family. Delineation of the family is mainly determined by phylogeny of 16S rRNA gene.

## Data availability statement

The datasets presented in this study can be found in online repositories. The names of the repository/repositories and accession number(s) can be found in the article/Supplementary material.

## Author contributions

ZH and QL conceived the study. LM, WW, and QL conducted the experiments. AO proposed names, wrote, and checked etymologies. All authors contributed to the article and approved the submitted version.

## Funding

This work was supported by Quanzhou City Science & Technology Program of China (No. 2022C018R), National Infrastructure of Natural Resources for Science and Technology Program of China (NIMR-2021-9), and Natural Science Foundation of Fujian Province (2022 J011106).

## Conflict of interest

The authors declare that the research was conducted in the absence of any commercial or financial relationships that could be construed as a potential conflict of interest.

## Publisher’s note

All claims expressed in this article are solely those of the authors and do not necessarily represent those of their affiliated organizations, or those of the publisher, the editors and the reviewers. Any product that may be evaluated in this article, or claim that may be made by its manufacturer, is not guaranteed or endorsed by the publisher.

## Footnotes

^1^https://blast.ncbi.nlm.nih.gov/Blast.cgi

^2^https://github.com/najoshi/sickle

^3^https://github.com/dparks1134/CompareM

^4^https://www.ncbi.nlm.nih.gov/genome/

## Abbreviations

MCCC, Marine Culture Collection of China
KCTC, Korean Collection for Type Cultures
MCL, maximum composite likelihood
NJ, neighbor-joining
ML, maximum-likelihood
iTOL, Interactive Tree of Life
dDDH, digital DNA–DNA hybridization
ANI, average nucleotide identity
AAI, average amino acid identity
